# Sport motivation and doping in adolescent athletes

**DOI:** 10.1371/journal.pone.0205222

**Published:** 2018-10-04

**Authors:** Jiri Mudrak, Pavel Slepicka, Irena Slepickova

**Affiliations:** 1 Department of Pedagogy, Psychology and Didactics, Faculty of Physical Education and Sport, Charles University, Prague, Czech Republic; 2 Department of Kinanthropology and Humanities, Faculty of Physical Education and Sport, Charles University, Prague, Czech Republic; Qazvin University of Medical Sciences, ISLAMIC REPUBLIC OF IRAN

## Abstract

**Background:**

Although performance-enhancing drugs appear to be prevalent in adolescent sports, relatively little attention has been paid to why adolescent athletes decide to use these drugs. In this study, we examine doping among adolescents from a motivational perspective and explore how motivational variables, such as achievement goal orientations and the perceived self-determination of sports activities, may be related to moral attitudes, doping intentions and doping behavior in adolescents who participate in competitive sports.

**Methodology:**

The study included 1035 adolescents participating in competitive sports from all regions of the Czech Republic (mean age = 16.3 years). The respondents completed a battery of questionnaires assessing their achievement goal orientations (task, ego), sports motivation at various levels of self-determination (intrinsic motivation, external regulation, amotivation), moral attitudes toward sport competition (acceptance of cheating, keeping winning in proportion, attitudes toward doping), doping intentions and doping behavior. A structural equation model was used to test the relations among motivational variables, attitudes, intentions and doping behavior.

**Principal results:**

Our analyses indicated a good fit with the proposed model, which explained 59% of the variance in doping intentions and 17.6% of the variance in doping behavior. Within the model, task orientation was positively associated with intrinsic motivation and lower amotivation, whereas ego orientation was positively associated with extrinsic regulation and amotivation. Furthermore, intrinsic motivation was positively associated with keeping winning in proportion and negatively associated with acceptance of cheating and attitudes toward doping; the less self-determined forms of motivation showed opposite relationships. However, only the acceptance of cheating and attitudes toward doping were related to doping intention, which subsequently predicted doping behavior.

**Conclusions/Significance:**

The results provide further evidence that sports motivation represents a psychological variable that should be considered in anti-doping policies, programs, and interventions aimed at the adolescent population because motivation was linked to the doping-related attitudinal variables and also partially mediated the effect of achievement goal orientations in this regard. On the basis of these results, we may argue that the focus on intrinsic enjoyment, self-referenced criteria of success and self-improvement may be related to more negative attitudes toward doping and cheating, lower doping intentions and less frequent doping behavior, whereas the emphasis on competition, comparison with others and external motivation appear to be related to the opposite outcomes.

## Introduction

The abuse of performance-enhancing drugs (PEDs) represents a significant problem in both competitive and leisure sports. The use of PEDs violates the spirit of fair play [1] and represents a significant health concern because doping has been linked to a number of health issues, including cardiovascular, neurological, and psychiatric disorders [2–3]. The World Anti-Doping Agency [4] reported that approximately 1% of the tested samples from Olympic sports athletes and approximately 3% of the tested samples from non-Olympic sports athletes showed positive results for doping. However, these relatively low numbers contrast with the results of questionnaire surveys that suggest a much higher prevalence of doping: approximately 10%-15% of competitive and recreational athletes report past or current use of doping, with some studies suggesting even higher percentages [5]. (Doping represents an umbrella term encompassing PED use, blood doping, gene doping, etc. However, we use the term doping in the article to refer only to the use of PEDs.)

Adolescent athletes may be considered particularly vulnerable to the abuse of PEDs. From a health perspective, adolescent users are at high risk of the side effects of PEDs such as anabolic steroids [3, 6]. From a psychological perspective, adolescents are especially susceptible to social pressures and expectations regarding sports competition and physical appearance [7] and tend to participate in risky behavior with possible harmful long-term effects [8]. A large-scale international meta-analytic study [9] observed that approximately 3%-6.5% of boys and 1%- 2% of girls reported current or past use of anabolic steroids. Other national surveys have found that, depending on the methodology used, 2.1%-11% of adolescents reported past or current use of PEDs [10–14].

A number of behavioral and psychological factors have been related to PED abuse in adolescents. Adolescent users of PEDs report more positive attitudes toward doping, show higher levels of moral disengagement toward doping and perceive higher approval of doping abuse by other people [12, 15]. Adolescent users of PEDs also report lower self-confidence and lower status in their peer group [7] and experience higher levels of anxiety [16], more frequent depression [17], lower self-regulation [12, 15], and more frequent use of other addictive substances, such as alcohol, tobacco, and hard drugs [11, 13]. Adolescent users of PEDs also experience more frequent eating disorders [17] and engage in other types of risk behavior, ranging from school absences [11] to membership in violent groups [18].

Two major motivations for adolescents’ use of PEDs have been discussed in the literature. First, adolescents use PEDs because they strive for physical attractiveness [7], which appears to be an especially dominant motive among adolescent athletes not engaged in competitive sports [2, 14]. For example, Sas-Novosielski [14] observed that a majority of adolescent PED users predominately strove for a “better body” with the primary goal of gaining muscle and losing body fat. Although more than half of the participants reported side effects from the substances (such as acne, hair loss, depression, and sexual disorders), they insisted that they would continue to use PEDs to improve their physical appearance. Second, adolescent athletes use PEDs to obtain a competitive advantage and succeed in sports competition. It appears that a focus on victory and success in competition has become a dominant discourse even in youth sports, which has increased the incidence of problematic behavior such as cheating and doping [19]. Motivational orientations that emphasize competitive performance and “winning at all costs” have been related to positive attitudes toward doping as well as toward doping behavior [20–21]. Although adolescent athletes generally report negative attitudes toward doping, they sometimes admit that they would be willing to use PEDs to develop their professional athletic careers [22].

Numerous research studies have suggested that attitudes toward doping, intentions to dope and actual doping abuse are significantly influenced by sports motivation; i.e., the subjective reasons underlying why athletes participate in sports affect the decision to use PEDs [23–30]. However, there are some limits to current research on the relationship between sports motivation and doping. Despite a recent surge of interest in this research topic [23–28], studies focusing on the relationships between sports motivation, doping-related attitudes, intentions and behavior in adolescents remain limited. In addition, some of the studies have investigated relatively small samples in the context of selected sports [27, 31], further limiting the generalizability of the current findings. Finally, there have been calls for more thorough implementation of coherent theoretical frameworks in doping research that would enable a better understanding of the psychological processes underlying doping behavior in adolescence [25, 31]. To address these limitations, this study’s primary goal was to further explore the motivational perspective on doping in a large sample of Czech adolescent athletes participating in competitive sports. In this manner, we integrate some key ideas from the achievement goal theory, self-determination theory and integrative model of behavioral prediction [32–33], and we postulate a series of relationships among achievement goal orientations, sports motivation, sports-related moral attitudes, doping intentions and actual doping behavior. On the basis of these hypotheses, we formulate a structural model, which we test within the structural equation-modeling framework.

## Theoretical framework

To understand the complexity of the psychosocial influences that determine an intentional behavior, Fishbein and Capella [33] proposed hierarchical relationships among behavior, an intention to carry out the behavior and behavior-related attitudes. Those authors suggested that “any given behavior is most likely to occur if one has a strong intention to perform the behavior, has the necessary skills and abilities required to perform the behavior, and there are no environmental or other constraints preventing behavioral performance” [33]. From this perspective, behavior-related intention is the direct determinant of a behavior, and we should strive to understand the factors that influence how an individual’s intentions to carry out a behavior are formed. Numerous studies have shown that doping-related attitudes represent a significant predictor of doping intentions [24, 27, 29]. However, we may hypothesize that the key attitudes related to doping intentions may be broader in scope and include more general attitudes toward cheating and winning in sports competition [19, 27] because the doping represents an instance of a cheating behavior [27] and has been related to increased emphasis on competition and winning in youth sports [19]. The attitudinal variables may then be considered proximal predictors of doping intentions that mediate the effects of other distant variables, including motivational beliefs [21, 24–25, 27].

To further explore the mediating role of attitudes in the relationship between sports motivation and doping, we adopted two well-established theories of sports motivation in our research. These theories include the achievement goal theory [14, 34–37] and the self-determination theory [25, 27, 38]. Specifically, based on these sports motivation theories, we consider task and ego achievement goal orientations and different positions on the self-determination continuum, including intrinsic motivation, external regulation, and amotivation, as possible predictors of doping attitudes and sports-related moral attitudes that further predict doping intentions and behavior. Below, we provide an introduction to these sports-motivation theories and their possible associations with these attitudes, intentions and behavior.

Based on self-determination theory [38], we expected that motivational states characterized by different levels of self-regulation (i.e., intrinsic motivation, external regulation and amotivation) may have different effects on moral and doping-related attitudes and behavior [23–25, 27]. Self-determination theory suggests that people strive to fulfill several basic psychological needs, such as the needs for autonomy, inner organization and better relationships with others. These basic needs are predominantly manifested by “intrinsically motivated behavior” or behavior that people engage in for its own sake, such as for the enjoyment stemming from the activity itself. The other end of the self-determination spectrum is represented by “externally regulated behavior,” which people engage in for external reasons, such as obtaining a reward or avoiding punishment. The least self-regulated motivational state is “amotivation,” in which people perceive a lack of self-regulation and personal agency toward the behavior. On the basis of extensive research, Deci and Ryan [38] asserted that engagement in intrinsically motivated behavior (as opposed to extrinsically regulated or amotivated behavior) is related to a range of positive outcomes, such as better performance, better relationships, and a higher level of well-being. With regard to doping, researchers have found that motivational states with higher self-determination were negatively related to attitudes toward doping [25], doping intentions [24], and past doping use [23], whereas external regulation was associated with moral disengagement in sports situations [27] or positive attitudes toward doping [28]. Furthermore, several other studies found that less self-determined forms of motivation predicted antisocial moral values and a lack of sportspersonship in athletes, including acceptance of cheating and winning-at-all-costs attitudes [39, 40]. On the basis of this research, we may argue that athletes who experience low levels of self-determination in their sport participation are lacking in some of their basic psychological needs and may compensate for this deficiency with more positive doping-related attitudes, intentions and behavior.

By contrast to self-determination theory, which focuses on why people engage in an activity, another group of motivational theories explores how different people subjectively prefer different achievement outcomes. Achievement goal theory [34, 41–43] conceptualizes these achievement outcomes through the dichotomy of “success in comparison with past performance” and “success in comparison with others.” In this framework, a subjective preference for one of these two dimensions has been determined to have different effects on achievement-related beliefs, choices, intentions and behavior. Various authors proposed different terms for these two dimensions, such as task-ego [34] and mastery-performance [42] orientations. It is important to note that in the context of doping research, the 2x2 model of achievement goal orientation has been used [23–24] to distinguish between two dimensions: mastery x performance and approach x avoidance (i.e., striving for success versus avoiding failure). However, effects on doping intentions and behavior have been observed in the mastery/performance rather than the approach/avoidance dimension, with mastery-oriented athletes showing lower doping intentions and behavior [23]. In our research, we applied the more traditional distinction of task-ego, which has also been used in the doping research [14, 36]. These two dimensions appear to be relevant in the context of doping: a negative relationship has been identified between the orientation toward improving past performance (task, mastery) and doping-related intentions, attitudes and behaviors, whereas the orientation toward comparison with other people (ego, performance) generally showed the opposite relationships [14, 24, 35–36, 44]. It seems that “task”-related goals do not provide an incentive for doping because these types of goals may be achieved solely through practice, and task-oriented athletes may be not motivated to expose themselves to the health and social risks related to doping. Conversely, “ego”-related goals appear to be supportive of doping because the standards of performance based on other athletes are much more difficult (or even impossible) to achieve, and athletes are more motivated to use immoral or even illegal practices to reach these goals [35–36, 44].

We may argue further that a link exists between the achievement goal orientations and self-determination [38, 45–47]. Task and ego goal orientations may be seen as different interpretative frameworks that influence the ways in which athletes perceive their autonomy, competence and relatedness to others. In this way, these achievement goal orientations affect the degree to which the athletes perceive themselves as self-determined [45–47]. For example, Duda and her colleagues [46] provided evidence that task orientation (as opposed to ego orientation) predicted sports-related intrinsic motivation in youth athletes. These authors argued that task orientation reduced the probability that athletes would perceive themselves to be incompetent because they compared themselves with self-referenced standards of achievement rather than standards set by other athletes, some of whom inevitably performed at a higher level. Similarly, other authors argued that task-oriented athletes experience fewer social constraints on their autonomy because they do not feel obliged to meet the performance standards set by other people and also experience better relationships because they do not perceive themselves to be in competition with others [45–47].

## Aim of the study

Based on the theoretical framework introduced above, we formulated a set of hypotheses regarding the relationships among the constructs of achievement goal orientation, self-determined sport motivation, attitudes, intentions, and doping behavior. We empirically tested these hypotheses within the structural equation modeling framework on a large sample of Czech adolescents involved in competitive sports. The implemented structural model was based on the following hypotheses:

H1: Task orientation is positively related to intrinsic sports motivation and negatively related to less self-determined forms of sports motivation (amotivation, extrinsic motivation).
H2: Ego orientation is positively related to less self-determined sports motivation (amotivation, extrinsic motivation) and negatively related to intrinsic motivation.
H3: Intrinsic motivation is negatively related to attitudes toward doping and acceptance of cheating and positively related to attitudes toward keeping winning in proportion.
H4: External regulation and amotivation are positively related to attitudes toward doping and acceptance of cheating and negatively related to attitudes toward keeping winning in proportion.
H5: Attitudes toward doping, acceptance of cheating, and keeping winning in proportion are directly related to doping intentions.
H6: Doping intentions are directly related to doping behavior.

## Methods

### Design of the study

The present paper is a component of the research project “Doping in Czech adolescents: Prevalence, correlates and experiences,” which was conducted with the support of the World Anti-Doping Agency. The data were collected from November 2014 –May 2015. The main part of the data collection occurred at high schools and elementary schools throughout the Czech Republic. The data collection at schools was facilitated by the Czech Association of School Sport Clubs, a nationwide educational organization that works with children and adolescents who are engaged in sports. Additionally, competitive and elite adolescent athletes were contacted through various Czech sports associations. In total, 60 schools and 7 sports associations participated in the research. Based on the preferences of the schools and sports associations, the questionnaires were administered either in paper form or by identical electronic questionnaires. The questionnaires were administered at schools or at training camps of the sports associations by the research team members and research assistants, who were PhD students of the Faculty of Physical Education and Sport, Charles University. Before the beginning of the data collection, the research was approved by the ethics committee of the Faculty of Physical Education and Sport, Charles University. The data collection was voluntary and anonymous, and the questionnaire was constructed in a way that prevented the identification of individual schools or respondents. Because the questionnaires were collected at the schools during school hours, the response rate was high (95%). Prior to the data collection, the children’s parents/guardians were informed of the research by the assisting teachers/coaches and requested to provide written consent. Approximately 5% of the contacted students did not participate in the data collection either because the parents/guardians did not provide consent or because the students were not willing to participate; these students were provided with an alternative activity under the supervision of the assisting teachers/coaches during the data collection.

### Sample

In total, we collected fully completed questionnaires from 2851 respondents (mean age 16.2 years, SD = 1.84). However, in the present article, we based our findings only on participants involved in competitive sports (n = 1035). The description of the sample is provided in Table 1.

**Table 1:** Demographic description of the respondents.

Demographic variable		
Age (years)	M (SD)	16.3 (1.92)
Gender	Male	64.4%
	Female	35.5%
Type of school	Elementary	32.6%
	Vocational	2.4%
	Secondary	42.1%
	Grammar	22%
	Other	.9%
Attending a sports school	Yes	25.1%
	No	74.9%

### Measures

In the first section of the questionnaire, the respondents were asked about demographic variables such as gender, age, and type of school and about their participation in sports (see Table 1). In the following section, the respondents were asked about their experiences with doping. The World Anti-Doping Agency defined doping as “breaking one or more anti-doping rules,” meaning that athletes who were found to be “positive” either used substances or methods present on the list of banned substances or were not compliant with doping control regulations [48]. On the basis of the WADA definition, some studies have examined the prevalence of doping by inquiring about the substances respondents used in the past that were subsequently classified according to the WADA list [15]. However, we deemed this approach appropriate for the population of adult athletes but not for adolescents. For the purpose of our study, we defined doping in the questionnaire as the “use of any substance which aims to enhance sport performance artificially and unfairly.” Therefore, we explored subjective evaluations of the respondents’ experiences with doping; i.e., we measured the extent to which the participants *believed* they doped to gain an unfair competitive advantage rather than measuring actual doping behavior. The respondents evaluated the frequency of their perceived experiences with doping on a six-point scale ranging from 1 (no) to 6 (yes, regularly). Similar research methods for doping prevalence have been implemented by other studies [13].

To assess the respondents’ attitudes toward doping, we used the Performance Enhancement Attitude Scale (PEAS) [49]. The PEAS is a one-dimensional 17-item scale measuring general attitudes toward doping in sports (unrelated to personal intentions to use doping). In the PEAS, respondents indicate on a 6-point Likert scale ranging from 1 (“completely disagree”) to 6 (“completely agree”) their agreement with statements evaluating various aspects of doping, such as “Doping is not cheating since everyone does it,” “Athletes are pressured to take performance-enhancing drugs,” or “The risks related to doping are exaggerated.” In scoring the PEAS, the overall score is obtained as the mean of all items. Overall, the PEAS shows good psychometric properties [47] and has been used in studies focusing on adolescent populations [15]. In our study, the PEAS showed acceptable reliability (Cronbach’s alpha = .755), although it did not originally fit well with our model. Confirmatory factor analysis of the one-dimensional scale showed poor fit indices (RMSEA = 0.075; 90% CI [0.069 to 0.081]; CFI = 0.826). We performed exploratory factor analysis of the scale that suggested 4 factors, which we labeled “Necessity of doping,” “Harmlessness of doping,” “Recreational drugs as doping,” and “Doping in media.” The four-factor model was further supported by confirmatory factor analysis that showed good fit (RMSEA = 0.054; 90% CI [0.047 to 0.060]; CFI = 0.926). In the structural equation model, we used a 7-item shortened version of the PEAS that included only items with factor loadings in the first factor higher than .5 (i.e., items 1–2, 6, 8, and 13–15 from the original scale).

To measure doping intentions, we utilized four items from an older Czech study focusing on the doping intentions of Czech adolescents [50]. The respondents answered on a scale ranging from 1 (“definitely not”) to 6 (“definitely yes”) regarding whether they would use doping in four hypothetical situations: 1) “Would you use doping if you strove for an important victory and were absolutely certain that nobody would find out?” 2) “Would you take a performance-enhancing substance that is not illegal but could have undesirable health effects?” 3) “Would you use doping if you were certain that it would help you succeed and would not have undesirable health effects?” and 4) “Would you use doping to enhance your performance if you knew that it would help you to achieve the highest level of sports success, such as winning the Olympic games?” On this basis, we computed doping intention as the mean of these four items. This scale showed good reliability (Cronbach’s alpha = .873). Furthermore, we used the Acceptance of Cheating and Keeping Winning in Proportion scales from the Attitudes to Moral Decisions in Sport Questionnaire [51] to measure general moral attitudes in sports situations. On these scales, respondents are asked to indicate on a 5-point Likert scale (from 1 –strongly agree to 5 –strongly disagree) how much they agreed with statements presenting them with sports situations, including a moral dilemma such as “It is OK to cheat if nobody knows?” or “Winning and losing are a part of life.” The Acceptance of Cheating scale appeared to have good reliability (Cronbach’s alpha = .895); the Keeping Winning in Proportion scale also had acceptable reliability (Cronbach’s alpha = .746).

To assess motivation-related constructs, we used selected scales from two questionnaires: the Perception of Success Questionnaire (PSQ) [52] and the Sport Motivation Scale-6 (SMS-6) [53]. The PSQ measures achievement goal orientations on a 5-point Likert scale, i.e., which types of sport-related outcomes are perceived as “success” by the respondents. The PSQ stems from the two-dimensional approach to achievement goal orientations and distinguishes between success stemming from mastering a task (“task”) and success in comparison with others (“ego”). Respondents indicate on a scale ranging from 1 (“strongly disagree”) to 5 (“strongly agree”) the sports situation in which they perceive themselves to be most successful. An example of the “task” item is “When playing sport, I feel most successful when I really improve,” and an example of an “ego” item is “When playing sport, I feel most successful when I am the best.” The “task” and “ego” dimensions are computed as the means of all corresponding items. Additionally, the PSQ showed good reliability in our study (Cronbach’s alpha = .857).

To measure the reasons why respondents participate in sports, we implemented several constructs based on self-determination theory [54]. Specifically, we used the dimensions of intrinsic motivation, external regulation, and amotivation from the SMS-6 [53], which represents a revised version of the Sport Motivation scale [54]. Each of these dimensions was measured by four items on a 5-point Likert scale ranging from 1 (“Does not correspond at all”) to 5 (“Corresponds completely”). Respondents were prompted by the statement, “Using the scale below, please indicate to what extent each of the following items corresponds to one of the reasons for which you are presently practicing your sport”, based on which the respondents indicated their reasons for their participation in sports. The items used to measure each dimension included “For the excitement I feel when I am really involved in the activity” (intrinsic motivation), “Because it allows me to be well regarded by people I know” (external regulation), and “I don’t know anymore; I have the impression of being incapable of succeeding in this sport” (amotivation). The SMS-6 questionnaire showed good psychometric properties (Cronbach’s alpha = .888 in our sample) and has been widely used in sports psychology research [53]. All scales and items used are provided in S1 Appendix.

### Analysis

We tested the hypothesized relationships within a structural equation modeling (SEM) framework using the statistical open source software R [55] and Lavaan, an R structural equation modeling package [56]. Only data from complete questionnaires were included in the analysis; therefore, there were no missing values. No outliers were identified in the data, and all the reported coefficients from our analyses were standardized. The model fit was assessed using standard measures of model fit: the chi-square statistic and corresponding p-value; the standardized root mean square residual (SRMR, which should approximate or be less than .08 for a good-fitting model) [57]; the root mean square error of approximation (RMSEA, with values approximately .05 or less being indicative of a close fit and values of .08 or less being indicative of a good fit) [58]; and the comparative fit index (CFI, in which values should be higher than 0.90 for adequately fitting solutions) [59].

## Results

Descriptive statistics and correlations of all variables included in the analysis are reported in Table 2. We observed significant but rather weak correlations among the majority of the variables included in the analyses. There were moderate to strong correlations among the motivational variables, such as task orientation-intrinsic motivation (r = .396) and ego orientation-external regulation (r = .315). Additionally, we observed strong correlations between doping intention and attitudes toward doping (r = .513) and between doping intention and acceptance of cheating (r = .663).

**Table 2:** Descriptive statistics and correlations.

	1. Task orientation	2. Ego orientation	3. Intrinsic motivation	4. External regulation	5. Amotivation	6. Winning in proportion	7. Acceptance of cheating	8. Attitudes toward doping	9. Doping intention	10. Doping behavior
1.	-									
2.	.341**	-								
3.	.396**	.237**	-							
4.	.110**	.315**	.460**	-						
5.	-.103**	ns	.ns	.259**	-					
6.	.259**	ns	.190**	-.091**	-.165**	-				
7.	-.125**	.243**	ns	.230**	.346**	-.197**	-			
8.	ns	.132**	ns	.135**	.204**	-.107**	.467**	-		
9.	ns	.250**	ns	.123**	.187**	-.132**	.663**	.513**	-	
10.	ns	.100**	.084**	.210**	.207**	-.107**	.346**	.265**	.391**	-
M	4.33	3.66	3.45	2.65	1.73	3.88	2.01	2.40	2.43	1.18
SD	.60	.80	.85	.94	.81	.83	.88	.78	1.15	.639

** Correlation significant at p < .001 level.

ns—correlation not significant

**Fig 1:**
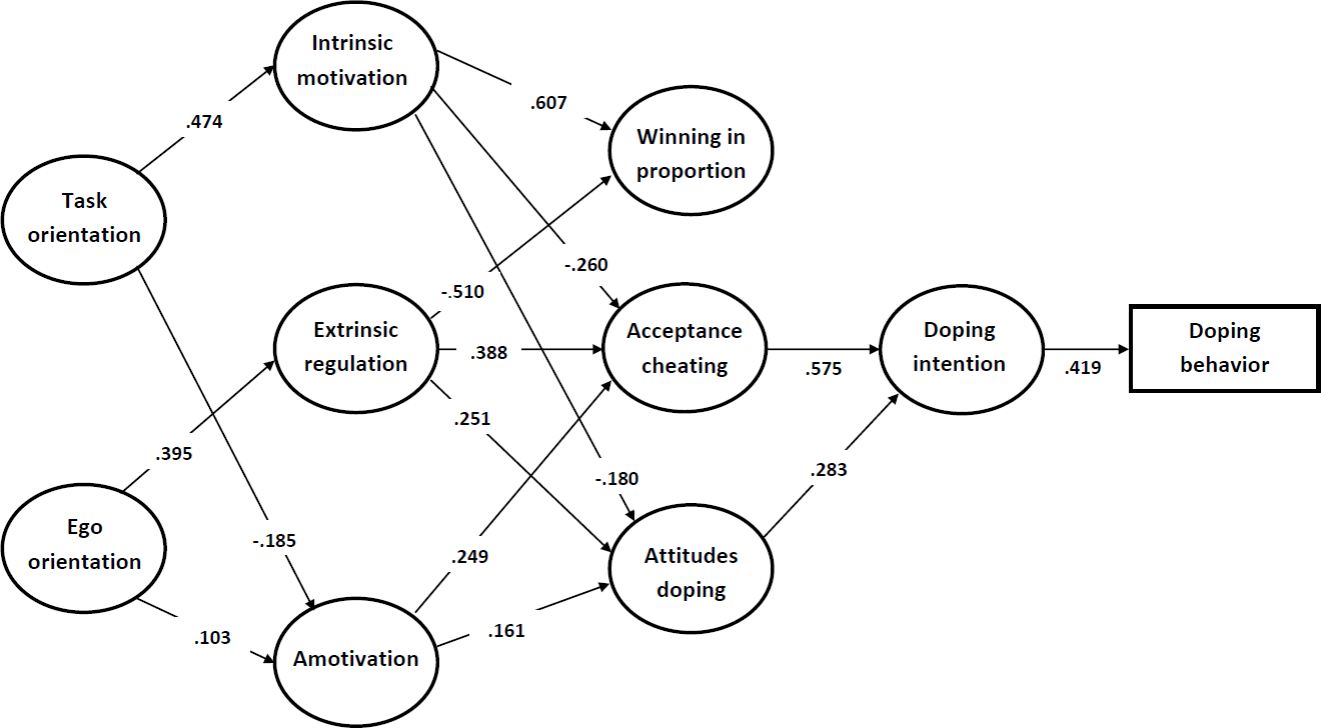
SEM model.

Based on our hypotheses, we formulated a structural model in which achievement goal orientations (task and ego) predicted sport motivation at different levels of self-determination (intrinsic motivation, extrinsic regulation and amotivation). The sports motivation was then associated with moral- and doping-related attitudes (attitudes toward doping, acceptance of cheating, and keeping winning in proportion) that were further related to doping intentions, which subsequently predicted doping behavior. Overall, we determined that the SEM model fit well with our data (χ^2^ = 19789.3; df = 946; p < 0.001; RMSEA = 0.042; 90% CI [0.040 to 0.044]; SRMR = 0.055; CFI = 0.913) and explained 59% of doping intentions and 17.6% of doping behavior. The measurement loadings for all latent variables were moderately high to high (range, 0.419–0.895) and highly significant (p < .001). The SEM model is presented in Fig 1. All measurement loadings are provided in S1 Appendix.

In accordance with our hypotheses, we observed that task and ego goal orientations were inversely related to sports motivation variables. Task orientation was linked to more self-determined sports motivation because it was moderately to strongly positively related to intrinsic motivation (β = .474) and negatively related to amotivation (β = -.185). At the same time, ego orientation was moderately to strongly positively related to extrinsic regulation (β = .395) and to amotivation (β = .103). Within the SEM model, the sports motivation was further associated with the attitudinal variables. Specifically, intrinsic motivation was negatively related to attitudes toward doping (β = -.180) and acceptance of cheating (β = -.260) and strongly and positively related to keeping winning in proportion (β = .607). Extrinsic regulation was positively associated with acceptance of cheating (β = .388), attitudes toward doping (β = .251), and negatively associated with keeping winning in proportion (β = -.510). Amotivation was positively associated with acceptance of cheating (β = .249) and attitudes toward doping (β = .161). The sports motivation variables also appeared to moderate the indirect effects of achievement goal orientation on the attitudinal variables. Specifically, we observed a negative indirect effect of task orientation on the acceptance of cheating (β = -.169), attitudes toward doping (β = -.115), and positive indirect effects on keeping winning in proportion (β = .288). In ego orientation, we observed a reversed direction of relationships because ego orientation was indirectly and positively related to the acceptance of cheating (β = .179), attitudes toward doping (β = .116), and indirectly and negatively related to keeping winning in proportion (β = -.202). Furthermore, the acceptance of cheating and attitudes toward doping were moderately to strongly related to doping intention, with acceptance of cheating having approximately twice the effect (β = .575) of attitudes toward doping (β = .283). Notably, keeping winning in proportion was not significantly related to doping intention. This suggests that excessive focus on winning may not be as important for the occurrence of doping behavior as athletes’ positive attitudes toward doping and cheating in sports. Finally, doping intention was moderately to strongly related to doping behavior (β = .419) within the SEM model.

## Discussion

As we hypothesized, the proposed model showed a good fit, and the observed relationships largely confirmed our expectations regarding the possible effects of sports motivation variables on doping attitudes, intentions and behavior in adolescent athletes. From the model, we may infer several main findings. First, doping intention may be perceived as an important predictor of doping behavior in competitive adolescent athletes, although not a perfect predictor. Overall, our model explained nearly the same amount of variance in doping behavior as an aggregate model based on meta-analysis of studies stemming from the theory of planned behavior, in which doping intentions were also used as the proximal predictor of doping behavior [5]. As argued by Fishbein [33], behavior-related intentions are particularly predictive of behavior when people have an opportunity to engage in the behavior and no environmental constraints are present. This is, of course, not a case of doping in adolescent athletes because this group can be expected to have limited access to doping and also perceive severe penalties related to doping. Therefore, we may argue that other variables also should be considered to explain doping behavior; simultaneously, however, doping intentions represent an important factor that should be targeted in doping prevention [24, 27, 29].

Second, a large portion of the variance in doping intentions was explained by the attitudinal variables included in the model. By contrast to the studies focusing solely on attitudes toward doping [12, 15, 24–25, 28–29, 31, 60], we broadened our scope and also included attitudes toward winning and cheating in sports as complementary attitudinal variables. These additions appeared to be particularly productive in the case of the acceptance of cheating. Hodge et al. [27] suggested that doping should be approached as an instance of cheating behavior; our findings corroborate this idea because the association between the acceptance of cheating and doping intentions was more than twice the size of the attitudes toward doping–the doping intention relationship. In any case, we may see both of these attitudinal variables as related, which has been supported by our results as well as by other studies [60]. Preventive anti-doping programs frequently target attitudes toward doping [61–63], and our findings suggest that focusing on broader moral values rather than on doping-specific attitudes may represent a more effective manner of understanding and preventing doping behavior. However, contrary to some authors, who suggested that the growing focus on winning in contemporary youth sports leads to more frequent occurrences of doping [19], the attitudes toward winning (i.e., keeping winning in proportion) did not show a significant relationship with doping intentions in the model. On this basis, we may argue that the doping may stem not from the focus on winning itself but rather from the growing acceptance of cheating and more positive attitudes toward doping that may be related to contemporary trends in (youth) sports [63].

Third, the intrinsic motivation showed negative relationships between attitudes toward doping and acceptance of cheating whereas the less self-determined forms of motivation showed relationships moving in the opposite direction. On the basis of the self-determination theory, we may argue that athletes who engage in competitive sports for enjoyment have satisfied through sports their “basic needs” of autonomy, competence and relatedness [38]. Therefore these athletes may place less value on the behavior that would provide them with further unfair advantages, such as doping or cheating. Conversely, athletes at a lower level of self-determination who experience a lack in some of these basic needs could be expected to have less restraint and demonstrate more positive attitudes toward these undesirable practices [39]. Similar results were reported by other authors: Zucchetti et al. [28] found that extrinsic motivation was related to more positive attitudes toward doping, and Chan et al. [25] observed that autonomous motivation predicted doping avoidance-related attitudes and, indirectly, the intention to avoid doping. Barkoukis et al. [23] determined that athletes high in amotivation reported higher doping intentions and higher past PED use whereas athletes high in external regulation reported higher past use of PEDs compared with other athletes. Therefore, we may argue that the positive effects of self-determined motivation [38] apply also to doping-related attitudes, intentions and behavior and that sports environments supporting such a positive motivational climate should be endorsed as a component of anti-doping efforts.

Fourth, sports motivation also mediated the effect of achievement goal orientations on the attitudinal variables within the model. Significant effects of achievement goal orientations on doping-related variables have been observed in numerous other studies: Barkoukis et al. [23] observed that athletes who emphasized mastery goals and de-emphasized performance goals also reported the lowest levels of past doping use and the lowest doping intentions. Sas-Nowosielski and Swiatkowska [36] determined that athletes with high task and low ego orientation reported the most negative attitudes toward doping whereas athletes with low task/high ego goals reported the most positive attitudes toward doping. These contradictory effects of mastery and performance orientations were also observed with regard to cheating and cheating intention in sports situations [64]. Our results suggest that these effects may be partially mediated by the relation between achievement goal orientations and sports motivation. Consistent with other researchers [45–47], we argue that a subjective preference of task or ego-related goals structures the experiences of athletes in a manner that affects the degree to which they perceive their sports participation as self-determined. Being “task oriented,” i.e., focusing on self-improvement and self-referenced standards of achievement, allows for disregarding societal constraints, comparison and competition with other athletes, which may be beneficial for the fulfillment of the basic needs of autonomy, competence and relatedness. However, “ego-oriented” athletes who set their standards of achievement based on the results of others may more easily question their competence, feel controlled in their sporting activity or experience worse relationships with other athletes, which affect the level of their self-determination. Because the sports motivation variables appear to be directly related to attitudes toward doping and cheating, we argue that the achievement goal orientations are related to doping intentions and behavior by this path.

Our study has some limitations that should be considered. Most importantly, the study employed a cross-sectional design that limits causal interpretations of the proposed relationships. We based our hypotheses on a review that suggested that the proposed direction of relationships would be at least partially valid; however, it is necessary to acknowledge that these relationships may be bi-directional, and we must interpret our results with caution. In addition, we included variables in the study that we hypothesized were important to doping in adolescents; however, a number of other variables that were not included may have similar or even greater effects. Additionally, although we recruited a large number of respondents from all regions of the Czech Republic and the response rate was high, our sample differed in some attributes from the general population of Czech adolescents. However, we believe that the sample showed sufficient diversity for the performed SEM analyses. We should also emphasize that we did not use objective methods to evaluate the prevalence of doping; instead, we relied on participants’ self-reports. Although self-reports of doping prevalence have been commonly used in studies of doping in adolescents [13], these methods may have significant limitations [64]. For example, respondents may perceive substances that are not on the list of banned substances to be doping, or they may conceal doping because it is generally a condemned behavior that may even lead to potential penalties. It is also important to note that the relationships between the sport motivation and doping-related variables were significant but weak-to-moderate in magnitude, which suggests that although motivational variables appear to play a role in doping among adolescents, this role should not be exaggerated. Finally, because our model explained a relatively low portion of variance in doping behavior, we may argue that the effect of sport motivation and attitudes toward doping/cheating is much more noticeable with regard to doping intentions than actual doping behavior. Other variables not included in our study, such as the availability or affordability of doping [65], may moderate the relations among motivation, attitudes, and doping behavior.

## Conclusion

The present research makes theoretical as well as practical contributions. Theoretically, we used well-established constructs of sports motivation and tested their hypothesized relations with doping-related variables in a complex model, largely confirming our hypotheses regarding the possible effects of achievement goal orientations and self-determined sports motivation. The tested model suggests a series of relationships between sports motivation and doping-related variables that are partially modifiable. Our findings thus suggest practical implications that may be used in doping-prevention efforts. First, it seems that it would be useful to target both doping-specific attitudes and general moral attitudes to decrease doping intentions and perhaps doping behavior. Second, sports motivation appears to play a significant role in attitudes toward doping and cheating and consequently toward doping intentions and actual doping behavior. The dimensions of sports motivation related to intrinsic immersion in the activity appear to have beneficial effects whereas the less self-determined forms of sports motivation may have some undesirable effects with regard to doping. Third, our results also suggest that achievement goal orientations are related to different levels of self-determination in sports activities and through this path, also to moral attitudes. In this manner, self-referenced task-goal orientations focusing on self-improvement appear to be beneficial whereas ego-goal orientations toward competition and comparison with others seem to have some detrimental effects. Therefore, our results further support the suggestions of numerous authors [9, 19–21, 63, 66] that the values present in contemporary youth sports that emphasize high-level performance, success in competition and victory at all costs may have negative consequences, including a greater susceptibility to doping. Positive change could come from parents, coaches, and teachers as well as sports and educational organizations, which all co-create a motivational climate and provide feedback that shapes individual motivational orientations [39, 67]. On this basis, we should once again endorse the classic Coubertin motto that “the important thing is not winning but taking part; the essential thing is not conquering but fighting well”.

## Supporting information

S1 Appendix Questionnaire items and measurement loadings. (DOCX)
