# Supplementary material for: Sport motivation and doping in adolescent athletes
Source: PLoS One. 2018 Oct 4;13(10):e0205222. doi: 10.1371/journal.pone.0205222 (PMC6171920; doi:10.1371/journal.pone.0205222)
Supplement: S1 Appendix — (DOCX) [file pone.0205222.s001.docx]

| **Scale** | **Item** | **Measurement loading in SEM** |
| --- | --- | --- |
| **Attitudes toward doping** | Legalizing performance enhancements would be beneficial for sports | 0.631 |
|  | Doping is necessary to be competitive | 0.606 |
|  | Athletes are pressured to take performance-enhancing drugs | 0.419 |
|  | The media blows the doping issue out of proportions | 0.422 |
|  | Doping is an unavoidable part of competitive sport | 0.650 |
|  | Athletes often lose time due to injuries and drugs can help to make up the lost time | 0.505 |
|  | Doping is not cheating since everyone does it | 0.700 |
| **Acceptance of cheating** | It is OK to cheat if nobody knows | 0.796 |
|  | I would cheat if I thought it would help me win | 0.880 |
|  | If other people are cheating, I think I can too | 0.785 |
|  | I cheat if I can get away with it | 0.812 |
|  | I would cheat if I thought it would help the team win | 0.728 |
| **Keeping winning in proportion** | Winning and losing are a part of life | 0.619 |
|  | It is OK to lose sometimes because in life you don’t win everything | 0.772 |
|  | If you win properly it feels better than if you did it dishonestly | 0.494 |
| **Doping intention** | Would you use doping if you strove for an important victory and were absolutely certain that nobody would find out? | 0.849 |
|  | Would you take a performance-enhancing substance that is not illegal but could have undesirable health effects? | 0.594 |
|  | Would you use doping if you were certain that it would help you succeed and would not have undesirable health effects? | 0.895 |
|  | Would you use doping to enhance your performance if you knew that it would help you to achieve the highest level of sports success, such as winning the Olympic games? | 0.856 |
| **Amotivation** (Why do you practice sport?) | I don’t know anymore, I have the impression of being incapable of succeeding in this sport | 0.491 |
|  | I don’t know if I want to continue to invest my time and effort as much in my sport anymore | 0.769 |
|  | It is not clear to me anymore; I don’t really think my place is in sport | 0.587 |
|  | I don’t seem to be enjoying my sport as much as I previously did | 0.757 |
| **External regulation** (Why do you practice sport?) | Because it allows me to be well regarded by people that I know | 0.619 |
|  | For the prestige of being an athlete | 0.749 |
|  | For the material and social benefits of being an athlete | 0.712 |
|  | To show others how good I am at my sport | 0.683 |
| **Intrinsic motivation** | For the excitement I feel when I am really involved in the activity | 0.613 |
|  | Because I feel a lot of personal satisfaction while mastering certain difficult training techniques | 0.706 |
|  | For the satisfaction I experience while I am perfecting my abilities | 0.777 |
|  | For the pleasure of discovering new performance strategies | 0.621 |
| **Task orientation** (When playing sport, I feel most successful when) | I try hard | 0.606 |
|  | I really improve | 0.745 |
|  | I reach a target I set for myself | 0.717 |
|  | I overcome difficulties | 0.764 |
|  | I succeed at something I could not do before | 0.728 |
|  | I perform to the best of my ability | 0.676 |
| **Ego orientation** (When playing sport, I feel most successful when) | I beat other people | 0.670 |
|  | I am clearly better | 0.817 |
|  | I am the best | 0.811 |
|  | I do better than others | 0.659 |
|  | I accomplish something others cannot do | 0.668 |
|  | I show other people I am the best | 0.737 |
